# The Decline in Population

**Published:** 1919-05-17

**Authors:** 


### THE DECLINE IN POPULATION.

The recent announcement of an actual excess of deaths over births in this country has, even in these days, created some sensation. The ordinary newspaper reader, who takes a short view in everything, considers it an unmixed evil. The neo-Malthusian, on the other hand, is rather elated. And since the opinion of the former may be heard in any railway carriage, while that of the latter, though much more reasoned, is set forth in sources of information comparatively recondite (although Dr. Havelock Ellis did write in a Sunday newspaper a while back), it will be of advantage to recount the sum and substance of the neo-Malthusian's argument. He considers that his master's contention as to the world's population being destined to outstrip means of subsistence has never been controverted, and therefore desiderates artificial checks on increase of population to reinforce the natural ones. He points out that these natural checks are to our minds appallingly cruel, appallingly blind and wasteful. For instance, he will say, in place of letting the ordinary impecunious working-class couple beget six children, lose two of them in infancy, and finally rear two healthy, and two invalidish ones, why not teach them to produce only three, and to bring these up sound in mind and body, thus saving doctors' bills, funeral expenses, wear and tear of the parents, and a revolting suffering of innocent babes? All the energy spent on a clinical attack on poverty-caused disease would be much better devoted to prophylactic and sanitary measures. Again, he will ask, why swell these physically inferior populations, urban nine-tenths of them, when the final result is to be war for means of outlet, for raw materials, and even for food? It is a strong case. Not many years of residence in India are required to enable one to see bubonic plague avert famine, and its absence contribute to it. And yet there are objections which may rightly be made. Europe has recently suffered a severe depletion, a severe blood-letting, and the end is not yet. What if the next four years be like the last ones—will the hardiest neo-Malthusian preach artificial restriction of reproduction then? What, again, of the advances of science in increasing food supply? It is hard to set a limit to these. There is, too, the natural instinct, not obscured yet, and quite distinct from random procreation, to increase and multiply, and to hand down one's name to succeeding generations. There is the evident folly of allowing a neighbouring nation to outnumber one's own, of, as it were, being the first to go in for deliberate limitation of population. As against this there is the mortification of losing "nationals" to other countries. Defoe called England "the jakes of Europe," where the latter voided her outcast offal progeny. America has long taken England's place in this respect. Far be it from us to deny that the neo-Malthusian has his rejoinders to these objections. This war depletion, he will say, effects its object very badly. It takes off young healthy men and leaves the cripples and diseased. Above all, it leaves an unsatisfied complement of women, already made much larger than is desirable owing to modern industrialism producing a male mortality larger than the female one. The whole subject, when its future aspects and its practical bearings are taken into account is thus highly complex. The proper attitude thereto is one of reserve and suspense. Nothing is less suited to treatment by (although not perhaps in) our present-day tawdry press. It is a case of getting more knowledge, more data; of listening only to those who have studied and reflected, and ignoring the men who speak from feeling only, or just for effect. And after that the experts will have to strike a balance between themselves, most likely an interim one. The moral is to be found in a motto inscribed below a figure of Darwin:

*Ignoramus: in hoc signo laboremus.*

